# Running out of time: the decline of channel activity and nucleotide activation in adenosine triphosphate-sensitive K-channels

**DOI:** 10.1098/rstb.2015.0426

**Published:** 2016-08-05

**Authors:** Peter Proks, Michael C. Puljung, Natascia Vedovato, Gregor Sachse, Rachel Mulvaney, Frances M. Ashcroft

**Affiliations:** Department of Physiology, Anatomy and Genetics, University of Oxford, Parks Road, Oxford OX1 3PT, UK

**Keywords:** K_ATP_ channel, rundown, MgADP activation

## Abstract

K_ATP_ channels act as key regulators of electrical excitability by coupling metabolic cues—mainly intracellular adenine nucleotide concentrations—to cellular potassium ion efflux. However, their study has been hindered by their rapid loss of activity in excised membrane patches (rundown), and by a second phenomenon, the *d*ecline of *a*ctivation by *M*g-*n*ucleotides (DAMN). Degradation of PI(4,5)P_2_ and other phosphoinositides is the strongest candidate for the molecular cause of rundown. Broad evidence indicates that most other determinants of rundown (e.g. phosphorylation, intracellular calcium, channel mutations that affect rundown) also act by influencing K_ATP_ channel regulation by phosphoinositides. Unfortunately, experimental conditions that reproducibly prevent rundown have remained elusive, necessitating *post hoc* data compensation. Rundown is clearly distinct from DAMN. While the former is associated with pore-forming Kir6.2 subunits, DAMN is generally a slower process involving the regulatory sulfonylurea receptor (SUR) subunits. We speculate that it arises when SUR subunits enter non-physiological conformational states associated with the loss of SUR nucleotide-binding domain dimerization following prolonged exposure to nucleotide-free conditions. This review presents new information on both rundown and DAMN, summarizes our current understanding of these processes and considers their physiological roles.

This article is part of the themed issue ‘Evolution brings Ca^2+^ and ATP together to control life and death’.

## 1. Introduction

K_ATP_ channels couple the metabolism of the cell to its electrical activity and thereby play important physiological roles in multiple tissues [1,2]. In pancreatic β-cells, for example, they couple the blood glucose concentration to insulin secretion, in neurones they regulate transmitter release, and in the cardiovascular system they contribute to vascular tone and the response to cardiac ischemic stress. The channel is an octameric complex of 4 pore-forming Kir6.2 subunits and 4 regulatory sulfonylurea receptor (SUR) subunits. Both subunits participate in metabolic regulation of channel activity: ATP binding to Kir6.2 closes the channel, whereas MgADP binding (or MgATP binding and hydrolysis) to SUR enhances channel activity [3–6].

An infuriating characteristic of K_ATP_ channels (at least from the perspective of the experimenter) is that their activity declines, seemingly inexorably, following patch excision into nucleotide-free solution. This property is known as rundown and is shared with all Kir channels and many other ion channels. It reflects the loss of one or more important physiological regulators, crucial for K_ATP_ channel function, upon patch excision. Exactly what causes K_ATP_ channel rundown, and how it can be prevented has been the topic of many investigations. The prevailing view is that it represents the loss of regulation by the membrane phospholipid phosphatidylinositol bisphosphate (PIP_2_), but other explanations have been also posited. Furthermore, no means of preventing rundown that is robust and translates from one laboratory to another has been identified. Indeed, even in the same laboratory, the speed and extent of rundown may vary from patch to patch or between cell preparations.

**Figure 1.**
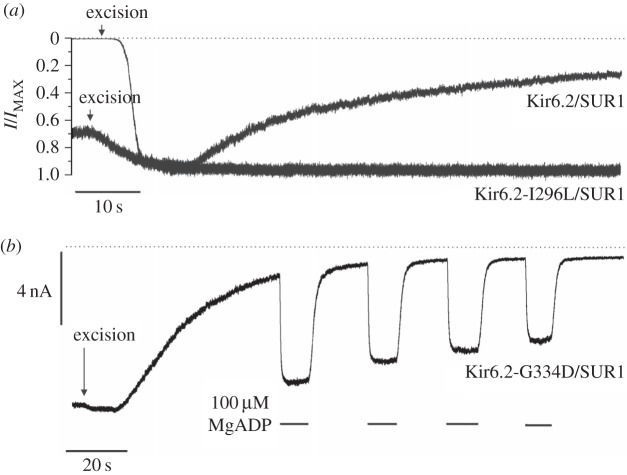
Rundown and decline of MgADP activation of K_ATP_ currents. (*a*) Representative recordings of macroscopic Kir6.2/SUR1 and Kir6.2-I296L/SUR1 currents at −60 mV in excised patches from *Xenopus* oocytes. For clarity of comparison, the currents are normalized to their maximal value after patch excision (*I*_MAX_). The dotted line indicates the zero current level and patch excision is marked with an arrow. The methods and solutions used are as described in [6]. (*b*) Representative recording of macroscopic Kir6.2-G334D/SUR1 current at −60 mV in an excised patch from *Xenopus* oocytes. Repetitive applications of 100 µM MgADP to the cytosolic side of the membrane are denoted by the bars. The dotted line presents the zero current level. The methods and solutions used are as described in [6].

Figure 1*a* illustrates the phenomenon. When the K_ATP_ channel is heterologously expressed in *Xenopus* oocytes no channel activity is observed prior to patch excision because the channels are almost fully blocked by the resting ATP concentration in the oocyte. Immediately following patch excision into nucleotide-free solution, channel activity increases as ATP is washed away from the intracellular surface of the membrane, reaching a peak within a couple of seconds. Subsequently channel activity runs down quasi-exponentially finally stabilizing at a level around 20% of maximal in this patch. Rundown is observed for all types of K_ATP_ channel including native β-cell and cardiac [7,8] channels, and recombinant Kir6.2/SUR1 and recombinant Kir6.2/SUR2 channels expressed in a variety of cell types [5,9,10].

Rundown is a problem for the researcher because measurements of channel ATP and MgADP/MgATP sensitivity are most easily performed in excised membrane patches. As a consequence, they are susceptible to errors induced by rundown and measures must be taken to reduce rundown or correct for it. Likewise, a second phenomenon—the decline of activation by Mg-nucleotides (DAMN)—that manifests in excised patches will influence studies of Mg-nucleotide activation (figure 1*b*).

Here, we review what is known about rundown and how it may be prevented. We consider how it may affect the outcome and interpretation of experiments, and review the extent to which its effects can be corrected. We demonstrate that rundown is distinct from the loss of Mg-nucleotide activation observed in excised patches, and we suggest a mechanistic explanation for the latter. We briefly also discuss how understanding rundown (and DAMN) provides important mechanistic insights into K_ATP_ function/regulation.

## 2. How does rundown affect the single-channel kinetics?

To assess the effect of rundown on K_ATP_ channel gating it is necessary to measure the single-channel kinetics both before and after rundown. As rundown is rapid and begins immediately on patch excision, the former is not possible in the excised patch. Ideally, therefore, the channel kinetics should be compared prior to patch excision and following rundown in the excised patch. However, this cannot be achieved for wild-type channels, which are strongly blocked in the cell-attached configuration by ATP present in the cell. We therefore used channels containing the Kir6.2-G334D mutation, which are almost completely insensitive to ATP inhibition [6,11], which activity is almost identical before and after patch excision (figure 1*b*), and which rundown with a time course identical to that of the wild-type channel [6].

**Figure 2.**
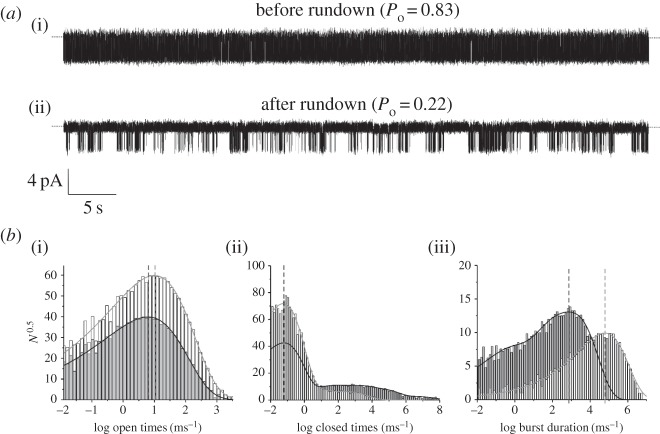
Effect of rundown on single-channel K_ATP_ channel properties. (*a*) Representative 1 min recording of single Kir6.2-G334D/SUR1 channels at −60 mV in the cell-attached configuration (*a*, *P*_o_ = 0.83) and after 5-min after patch excision (*b*, *P*_o_ = 0.22). (*b*) Distributions of channel open times (i), closed times (ii) and burst duration (iii) before (pale grey bars) and after (dark grey bars) rundown. The distributions were fitted with probability density functions that gave the following values for the individual components. Before rundown: mean open time, 2.7 ms; three apparent closed states with mean values of 0.3 ms (98.5%), 2 ms (0.8%) and 12 ms (0.7%); and a single burst state with a mean duration of 120 ms. After rundown: mean open time 2.2 ms; five apparent closed states with mean values 0.3 ms (88.9%), 3 ms (3.3%), 19 ms (4.3%), 82 ms (3.1%) and 650 ms (0.4%); and three apparent burst states with mean durations of 23 ms (66%), 5 ms (20.5%) and 0.5 ms (13.5%). Vertical lines indicate the mean values for open times, short closed times and the major burst duration component, and illustrate that rundown has no effect on the intraburst closed times, but reduces the mean open time and burst duration. The methods and solutions used are as described in [6].

Figure 2 shows that channel activity is characterized by long bursts of openings with both intra- and inter-burst closings. Prior to rundown there is a single burst state, a single open state and a single closed state within the burst (both with high occupancy), and two long interburst closed states that are entered infrequently. After rundown, occupancy of the intraburst open and closed states is substantially reduced. The mean open time is reduced by 20%, indicating rundown destabilizes the open state of the channel. The frequency, duration and apparent number of the inter-burst closed states also increases, implying rundown stabilizes the long closed states of the channel. The burst distribution now contains two additional short components (giving a total of three). Since SUR is known to increase burst duration [12], this may indicate that rundown destabilizes the interaction of SUR1 with Kir6.2.

## 3. Is sulfonylurea receptor involved in rundown?

Neither SUR1 nor Kir6.2 is correctly trafficked to the membrane in the absence of their partner subunit, owing to the presence of ER retention tags in both subunits [13]. However, deletion of the last 26–36 amino acids of Kir6.2 (Kir6.2ΔC) removes the retention tag enabling Kir6.2 to express at the plasma membrane in the absence of SUR [4,13]. Thus Kir6.2ΔC channels can be used to assess the effects of rundown on Kir6.2 alone. K_ATP_ channels composed of both Kir6.2 and SUR subunits, or Kir6.2ΔC alone, had similar rundown properties to wild-type channels [4,9]. Thus rundown is intrinsic to Kir6.2. The extent to which SUR influences rundown, if at all, has not been quantified. However, no clear, robust differences in rundown have been reported in the presence of either SUR1 or SUR2.

## 4. What causes rundown?

Mechanisms that have been proposed for rundown include loss of regulation by membrane phospholipids such as phosphatidylinositol 4,5-bisphosphate (PI(4,5)P_2_, here abbreviated as PIP_2_), dephosphorylation or proteolysis of the channel, and loss of interaction with cytoskeletal proteins. It is likely that several of these mechanisms operate in concert, with their relative contributions varying with the prevailing conditions. Here, we briefly summarise, in turn, what is known of each of these mechanisms.

### (a) Loss of phosphoinositide regulation

Anionic phospholipids (e.g. PIP_2_) activate all inward rectifier K^+^ (Kir) channels and degradation of phospholipids by endogenous lipid phosphatases or phospholipases is a well-accepted mechanism for Kir current rundown in excised membrane patches. The rate of Kir current rundown varies and is inversely correlated to the PIP-binding affinity of the channel being studied, with rundown being faster for channels that bind PIPs less strongly [14–16].

Sensitivity of K_ATP_ channels to phosphoinositide turnover was first demonstrated in giant membrane patches from cardiac myocytes, where native Kir6.2/SUR2A channels are abundant [17]. These channels run down rapidly in excised patches exposed to nucleotide-free solutions, but following exposure to intracellular MgATP their activity is (at least partially) restored, as seen by comparing the current in control solution before and after ATP application. This increase in channel activity was mimicked by intracellular application of PIP_2_, and reversed by exposure to phospholipase C (PLCβ) or Ca^2+^ (which presumably activates endogenous PLCs). Further, MgATP activation was prevented by prior application of a phosphoinositol-(PI)-specific PLC, suggesting that MgATP works through endogenous PI kinases. Thus, these data argue that rundown is due to loss of channel regulation by PIP_2_, and that regeneration of PIP_2_ restores channel activity. Similar activation by PIP_2_ has been demonstrated for native K_ATP_ channels in other tissues and for recombinant K_ATP_ channels [18–21]. Importantly, like rundown, phosphoinositides interact with Kir6.2, as evidenced by their ability to activate Kir6.2ΔC in the absence of SUR [10,20,21]. This suggests a conservation of mechanism across the Kir family.

Kir6.2-containing channels have a relatively broad specificity for phosphoinositides. In addition to PI(4,5)P_2_, they are activated by PI(4)P (PIP), albeit at higher concentrations [18,22]. In agreement with the finding that PIP does not activate the channel as strongly as PIP_2_, inhibition of PI(5)K (which phosphorylates PIP to PIP_2_) decreases the ability of MgATP to refresh Kir6.2/SUR2A channels expressed in HEK cells [23]. Activation of enzymes that degrade phospholipids [17,18,21], or application of polyvalent cations that chelate phosphoinositides to the intracellular membrane surface [18,20], leads to channel inhibition. Interestingly, activation of the voltage-sensitive lipid phosphatase CiVSP, which inhibits many other inward rectifier channels does not reduce K_ATP_ currents [22]. This is probably because CiVSP hydrolyses PIP_2_ to PIP, which still activates Kir6.2-containing channels.

As described above, rundown of K_ATP_ channels is associated with a decrease in the duration of the open and burst states and an increase in the frequency and duration of the inter-burst closed states. It is also accompanied by a very fast increase in sensitivity to ATP inhibition [10]; this effect is not always evident, however, presumably because of its rapidity. Activation by phosphoinositides has the reverse effect on the channel kinetics [19]. Phosphoinositides also antagonize the ability of ATP to inhibit K_ATP_ channels, whether native or recombinant [19–21]. They also reduce the ATP sensitivity of Kir6.2ΔC without dramatically affecting *P*_o_ [21].

**Figure 3.**
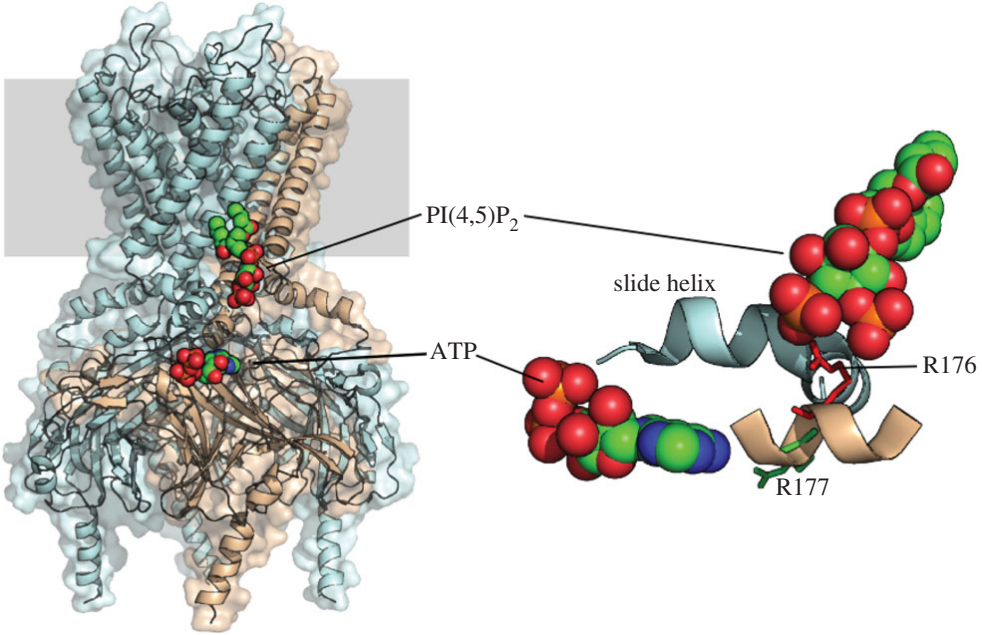
PIP_2_- and ATP-binding sites of Kir6.2. Left*.* Structural model of Kir6.2 based on the X-ray structure of Kir2.2 [25]. The PI(4,5)P_2_ molecule is positioned as in Kir2.2. A single subunit is shown in pink and the remaining three are blue. The membrane is shown in grey. The ATP is positioned based on the model by Antcliff *et al.* [26]. Right*.* A different view of the binding sites showing key PIP_2_-binding residues (R176 and R177, [18]), and the slide helix.

Some studies suggest that PIP_2_ affects ATP sensitivity by two mechanisms: an indirect effect resulting from the change in channel kinetics and a direct effect on ATP binding. In single Kir6.2ΔC channels, the magnitude of the change in ATP sensitivity produced by PIP_2_ is difficult to explain based on the reported increase in open probability alone [19]. However, this does not necessarily imply there are two separate binding sites for PIP_2_, as both effects could theoretically be accommodated by PIP_2_ interaction with a single site [24]. This question might be explored by analysis of the atomic structure of the channel in complex with PIP_2_. However, no high-resolution structure for the K_ATP_ channel has yet been reported. The atomic structure of the related Kir channel Kir2.2 in the presence and absence of short-chain PIP_2_ firmly establishes a conserved set of amino acids that form a PIP_2_-binding site and suggests a mechanism whereby lipid binding stabilizes the open state of the channel [25]. Figure 3 illustrates the proposed PIP_2_-binding site in Kir6.2, based on this structure. Mutations in residues that line the site (e.g. R176A, R177A) affect the phosphoinositide sensitivity of Kir6.2/SUR1, accelerate rundown and reduce the ability of PIP_2_ to attenuate ATP inhibition [14,18,21]. The putative PIP_2_ site is also close to the putative ATP-binding site [26] (figure 3). Several studies have suggested that PIP_2_ can displace ATP from its binding site, and *vice versa* [20,24,27,28], but as the sites appear to be structurally distinct this must be via an allosteric interaction or electrostatic repulsion.

In summary, there is considerable evidence in support of the idea that rundown is owing to loss of phosphoinositide regulation. It should be recognized that this does not necessarily mean PIP_2_ will dissociate from the channel, as dephosphorylation to PIP or PI *in situ* will also lower *P*_o_. It is also worth noting that rundown is associated with a decrease in *P*_o_, so any agonist (or mutation) that increases *P*_o_ will mask rundown. However, because rundown owing to phosphoinositide degradation is a common theme throughout the Kir family, it remains a strong candidate for the cause of K_ATP_ channel rundown. It should also be appreciated that channel regulation by PIP_2_ will help set the level of channel activity in the intact cell, and that agonists or antagonists that alter the membrane concentration of PIP_2_ will also affect K_ATP_ channel function [21,29].

Kir6.2-containing channels can also be activated by long-chain acyl-CoA esters which, like PIP_2_, reduce channel inhibition by ATP and slow rundown [15,30,31]. As long-chain acyl-CoAs act as competitive antagonists of PIP_2_, it is presumed that they interact with the same binding site [32]. To what extent channel activity in the cell is influenced by endogenous acyl-CoAs and how much their loss contributes to rundown remains unclear.

### (b) Gating mutations

A large number of mutations in both Kir6.2 and SUR1 have been shown to affect K_ATP_ channel kinetics [2]. ‘Gating’ mutations that result in a near-maximal open probability (e.g. I296L) also dramatically slow rundown (figure 1*a*). Within Kir6.2, they mainly reside in regions that are thought to move when the channel opens and closes [2], whereas in SUR1, they principally lie in TMD0 or the CL3 linker, regions that are well established to modulate the *P*_o_ of Kir6.2 [33,34].

It has been proposed that gating mutations exert their effect on the channel kinetics by strengthening the interaction of PIPs with Kir6.2 [35]. The ability of these mutations to slow rundown would then result from a greatly reduced off-rate for PIP binding to Kir6.2, which would attenuate PIP dissociation and prolong channel activity. The ability of TMD0 to enhance *P*_o_ (and slow rundown) has also been proposed to be mediated via enhanced PIP_2_ interaction with Kir6.2 [36]. Without binding data these ideas cannot be confirmed. However, most gating mutations in Kir6.2 lie well outside the proposed PIP_2_ binding site(s) on Kir6.2 and no PIP_2_ binding site has (yet) been identified in SUR1. Thus any effect of gating mutations on PIP_2_ binding must be allosteric. Alternatively, gating mutations may cause modifications in channel structure that lead to conformational states similar to those that are produced by PIP binding. This would also be expected to slow rundown.

Mutations at positions that are thought to be involved in inter-subunit interactions within Kir6.2 (e.g. E229, E227) cause rapid inactivation of K_ATP_ channels in excised patches [37,38]. It is not entirely clear whether this rapid inactivation is simply accelerated rundown or a different phenomenon. It has been argued that the inactivation seen for R314A and E229A is not identical to normal rundown as it occurs in the presence of EDTA, which in these experiments abolished rundown [37]. Rather, it was suggested inactivation arises from the loss of stabilizing interactions between an intra-subunit ion pair that, in the wild-type channel, facilitates the ability of other positively charged residues to interact with membrane phosphoinositides.

### (c) Dephosphorylation

Ohno-Shosaku *et al.* [7] first showed that application of MgATP to the inner membrane surface of an excised patch not only caused inhibition of β-cell K_ATP_ channel activity but also resulted in a marked increase in channel activity when ATP was subsequently removed. They attributed this ‘reactivation’ or ‘refreshment’ of channel activity to phosphorylation because ATP in the absence of Mg^2+^, and the poorly hydrolysable ATP-analogues AMP-PNP, AMP-PCP and ATPγS were ineffective. Refreshment is also observed for Kir6.2ΔC expressed in the absence of SUR, indicating that it is intrinsic to Kir6.2 [4].

While phosphorylation is clearly involved in refreshment, the key question is what gets phosphorylated—the channel itself, a regulatory protein or membrane lipids such as PIP_2_? Regulation of the K_ATP_ channel via phosphorylation of Kir6.2 has been reported for both protein kinase A and protein kinase C [39,40]. The former enhanced the channel open probability and the latter led to internalization of the channel. Effects on rundown were not reported in these studies. However, refreshment was not prevented by a range of protein kinase inhibitors, including inhibitors of PKA and PKC, making it unlikely protein phosphorylation is involved [41]. By contrast, the lipid kinase inhibitor wortmannin, which inhibits PI 3-kinase and (at higher concentrations) PI-4 kinase, abolished MgATP-dependent recovery of K_ATP_ channels inactivated by Ca^2+^ [41]. ATP failed to increase Kir6.2/SUR2A channel activity following degradation of PI by a specific PLC, but activation could be restored by exogenous application of PI, implying that phosphorylation of PI-based lipids was responsible [17]. This supports the idea that refreshment is due to PIP_2_ generation by lipid kinases.

### (d) Effects of cations

Exposure of native and recombinant cardiac K_ATP_ channels to intracellular solutions containing elevated Ca^2+^ (>100 µM) induces very rapid rundown that is reversed by MgATP [17,41,42]. Reactivation was blocked by the lipid kinase inhibitor wortmannin [41]. Nevertheless, the loss of channel activity provoked by Ca^2+^ could be reversed by PIP_2_ even after wortmannin treatment. Taken together, these experiments support the idea that Ca^2+^ causes rapid K_ATP_ channel closure by activating Ca^2+^-dependent lipid phospholipases (e.g. phospholipase C), leading to loss of membrane PIP_2_ and PIP; and that MgATP-dependent reactivation of channel activity is due to the rephosphorylation of regulatory phospholipids.

Whether lipid phospholipases/phosphatases are activated by the Ca^2+^ concentration in normal intracellular solutions is unclear, but as Ca^2+^ is normally buffered to very low levels with EGTA in electrophysiology studies and rundown still occurs, this seems unlikely. However, in some cases, the rate of rundown was indeed enhanced by the removal of EGTA from the intracellular solution [19]. It seems possible there may be a continual turnover of PIP_2_ in the membrane, with intrinsic phosphatase activity being balanced in the cellular environment by simultaneous kinase activity. In the absence of MgATP, this will lead to a steady decline in PIP_2_ concentration and K_ATP_ channel rundown.

It is also possible that Mg^2+^, or other divalent cations, accelerate rundown, because addition of 1 mM EDTA to the bath solution markedly slowed rundown of native β-cell K_ATP_ channels [43] and Kir6.2/SUR1 channels expressed in Cosm6 cells [37]. However, in our experience, while EDTA can be effective in preventing rundown of K_ATP_ channels expressed in mammalian cell lines this is not always the case for those expressed in *Xenopus* oocytes.

Exposure to low intracellular pH also causes an irreversible loss of K_ATP_ channel activity [44]. However, this process appears distinct from normal rundown, as it is not evident until the pH drops below 6.4, which is far less than the pH of intracellular solutions usually employed. Nevertheless, it is possible that low pH induces the same conformational state as rundown (or loss of PIP_2_).

### (e) Proteolysis

Patch excision has also been proposed to activate Ca- and/or Mg-dependent proteases that are normally inhibited in the intact cell. However, proteolysis seems unlikely to be the cause of normal rundown for several reasons. First, in general, rundown cannot be prevented by buffering the intracellular concentration of divalent cations to very low levels with EGTA, and EDTA (while effective in some cases [37,43], it is not always a panacea). Second, proteolysis by trypsin or papain actually prevents rundown of β-cell [45] or cardiac [46] K_ATP_ channels, rather than inducing it. This is probably because trypsinization irreversibly increases the channel open probability (*P*_o_) to approximately 0.8 [44,45], and channels with high *P*_o_ exhibit reduced rundown (see above). Proteolysis also removed MgADP activation and glibenclamide block [45,47,48], which is not observed when channels run down normally.

### (f) Loss of cytoskeletal interactions

Patch excision is not only associated with a change in the composition of the intracellular solution but also involves severance of cytoskeletal connections. Actin filament-depolymerizing agents (e.g. cytochalasin D and DNase1) accelerated rundown of native cardiac K_ATP_ channels [49]. Conversely, the actin filament stabilizer phalloidin inhibited both spontaneous and Ca^2+^-induced rundown. Interestingly, F-actin together with MgATP was able to restore rundown channels, even when MgATP alone could not.

Rundown is also observed in the open-cell-attached condition, in which the patch membrane is not disrupted and the channel presumably remains attached to the cytoskeleton [8,50]. Nevertheless, rundown is slower than in the excised patch: for example, the current decreases less than 10% in 6 min [8,50]. It is possible that perfusion with intracellular solution leads to activation of lipid phosphatases or inactivation of PIP_2_ regeneration. Finally, one should not forget that PIP_2_ influences actin cytoskeleton remodelling and membrane targeting of A-kinase anchoring proteins [16], and thus potentially might influence K_ATP_ channel activity via such secondary interactions.

## 5. The molecular basis of rundown: a synthesis and a hypothesis

Evidence summarized here supports the idea that the loss of PIP_2_ is the dominant factor responsible for rundown of K_ATP_ channel activity. Variability in endogenous PIP_2_ levels, and in the activity of endogenous phosphatases probably underlies differences in the rate of rundown between different cells and laboratories. While rundown of most Kir channels simply reflects dephosphorylation of PIP_2_, this is not the case for the K_ATP_ channel, which is also activated, but to a lesser extent, by PIP. PIP is approximately fivefold less potent at activating the K_ATP_ current than PIP_2_ [18]. As channel rundown often stabilizes at around 20% of the initial current magnitude, we speculate that this may represent channels with bound PIP. The secondary slow decline in channel activity from this pseudo steady-state level may reflect dephosphorylation of PIP to PI, which does not support K_ATP_ channel activity [18]. This idea may explain the biphasic time course of rundown (fast then slow) seen in many cells.

## 6. How can one prevent rundown or mitigate its effects?

In order to study K_ATP_ channel gating, it would be helpful to be able stabilize channel activity at some steady state, preferably one that approximates that in the intact cell. Numerous techniques have been proposed to slow rundown significantly, either in the literature or anecdotally. These include low-Mg^2+^ intracellular solution [43]; a cocktail designed to inhibit lipid phosphatases consisting of 5 mM F^−^, 10 mM pyrophosphate and 0.1 mM vanadate (FVPP solution [14]; gluconate rather than chloride as the main intracellular anion [51]; 1 mM EDTA [37], EGTA [18] and PIP_2_ itself [20,21]).

Unfortunately, in our hands, we have found nothing that routinely prevents rundown, and conversations with many other investigators suggest that this is also their experience. While some manipulations may appear to do so in certain cells or cell types, at random times of the year, or for certain combinations of recombinant K_ATP_ channel subunits, this is not always the case—and what is found in one laboratory does not necessarily translate to another.

It might be argued that rundown could be stabilized simply by adding a fixed concentration of PIP_2_ to the bath solution to produce a stable level of channel activity. However, this is very difficult to achieve because PIP_2_ continues to incorporate into the membrane following its addition, as it is highly hydrophobic. This explains why the effects of PIP_2_ application increase over time, as the lipid accumulates in the patch membrane [20,21]. A non-hydrolysable water-soluble analogue is needed. One possibility might be diC8-PI(4,5)P_2_, a water-soluble PIP_2_ analogue [15]; however, this is extremely expensive and thus cannot be used routinely for experiments. Furthermore, what level of channel activity corresponds to that in the cell is contentious, making it difficult to know how much diC8-PI(4,5)P_2_ should be used.

An alternative might be to use a gating mutation. However, this has the usual drawbacks of using a mutant channel: i.e. it is not known what other effects the mutation may have. Furthermore, because channels with gating mutations still rundown, albeit to a much lesser extent, their properties may also change with time in excised patches. Furthermore, most gating mutations that suppress rundown also strongly impair ATP inhibition. More promising might be K_ATP_ channels composed of tandem SUR1-Kir6.2 or Kir6.2-Kir6.2 subunits, which can produce channels with little rundown and relatively small shifts in ATP sensitivity (e.g. [52]). Nevertheless, these channels still have a high *P*_o_, which might not be optimal for some studies: e.g. if the aim of the experiment is to study channel activation.

This means that it is necessary to correct for rundown in most experiments, as it cannot be prevented. The traditional way to do so when constructing an ATP concentration–response curve is to bracket each ATP concentration with nucleotide-free solution, then to take the mean of the current in the control solutions on either side of the test ATP solution and then express the latter as a fraction of the former. However, this raises the question of whether value taken for the control current should be the peak current, the current at the end of control solution application, or the mean of the current averaged across the total application time. In practice, we find that it does not matter, as the dose–response is the same. But this should always be tested. In addition, a single test ATP concentration may be applied at intervals to check that the ATP sensitivity remains unchanged throughout the course of the experiment.

## 7. Oh DAMN

In addition to the decline in *NP*_o_ following patch excision (rundown), the ability of MgADP to stimulate channel activity through its interactions with the SUR subunit of the K_ATP_ channel also declines with time. This phenomenon has been termed the *d*ecline of *a*ctivation by *M*g-*n*ucleotides—DAMN [6]. It is a process distinct from channel rundown, as evidenced by the fact that channel activity can remain long after the ability of MgADP to enhance the channel open probability is lost. The time course of DAMN is very variable but it is normally complete within 30 min of patch excision and usually sooner [6,53]. Interestingly, the number of functional channels (*N*) appears to decline faster than the *P*_o_ [6].

What might underlie DAMN? Clearly one possibility is that SUR becomes functionally disconnected from Kir6.2. However, full dissociation cannot occur as the ability of SUR to enhance the ATP sensitivity of Kir6.2 does not change, and inhibition by sulfonylureas remains (at least partially) intact. It seems that DAMN is specific for Mg-nucleotide activation.

**Figure 4.**
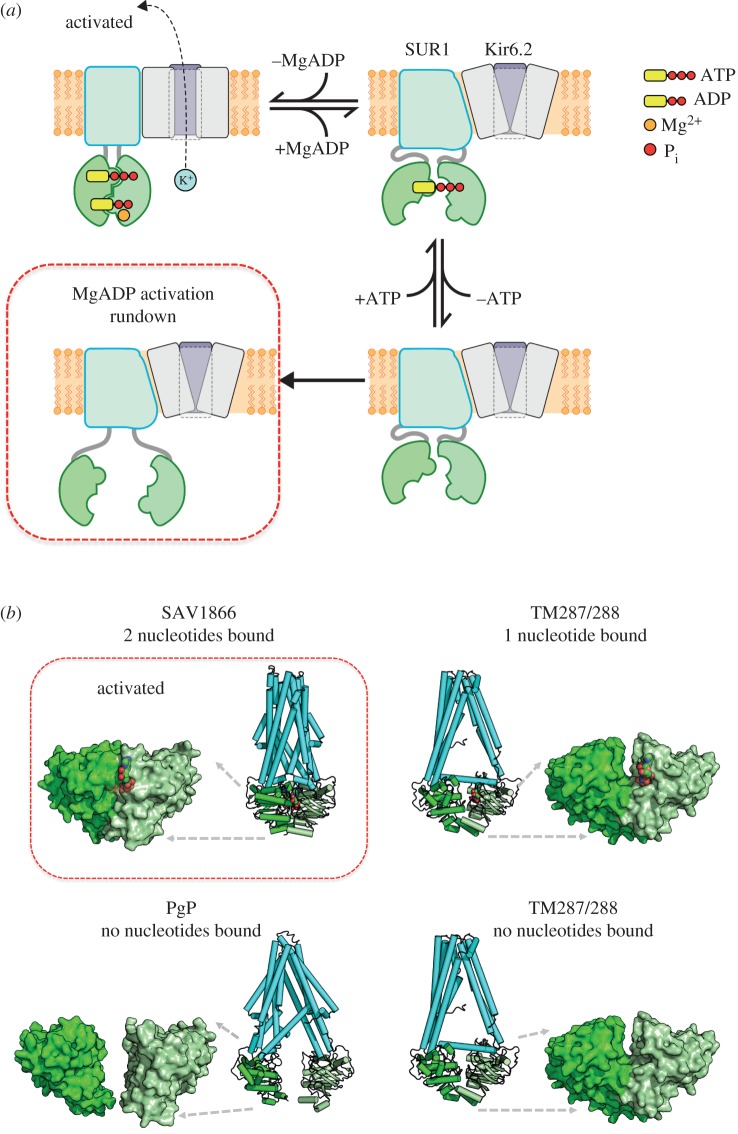
A structural model for DAMN. (*a*) Model of the SUR reaction cycle. A single SUR subunit is shown. (*b*) X-ray structures of SAV1866 [54], TM287/288 [55,56] and PgP [57] with/without bound nucleotide (red), as indicated. NBD1 bright green, NBD2, pale green, TMs cyan. Left, NBD dimer. Right, TMs plus NBDs.

We therefore speculate that DAMN results from the inability of the NBDs to dimerise, as illustrated in figure 4*a*. It is well established that the nucleotide-binding domains (NBDs) of ABC proteins associate to form two nucleotide-binding sites (NBS), each composed of the W_A_ motif and W_B_ motif of one NBD and the signature sequence of the other NBD, with nucleotides sandwiched at the interface [54,58]. There is also evidence that, like some other ABC proteins [55], SUR1 has two asymmetric NBSs. Known as the degenerate site, NBS1 binds ATP with high affinity but does not hydrolyse it [59]. Conversely, NBS2 is a consensus ATP-binding site that binds and hydrolyses MgATP. It is believed that ATP binding to NBS1, and MgADP binding at NBS2, of SUR1 leads to channel activation [60]. When nucleotides are removed, MgADP will dissociate from NBS2, leading to channel deactivation. Loss of nucleotide at NBS2 facilitates the subsequent unbinding of ATP from NBS1 [61].

We hypothesize that, initially, the conformation of the NBDs in the apo state is not very different from the states in which nucleotide is bound at one or both NBSs. As the NBDs remain close together, it is possible for nucleotide rebinding (and stimulation of channel activity) to take place if the intracellular membrane surface is once again exposed to Mg-nucleotides. After some time in nucleotide-free solution, however, we postulate that the lack of bound nucleotides results in complete dissociation of the NBDs. As a consequence, nucleotide binding and NBD dimerization is impaired. This state corresponds to the DAMN rundown state.

The states we postulate correspond to conformational states identified in ABC proteins in crystallographic studies (figure 4*b*). In Sav1866, where two nucleotides are bound, the NBDs are locked together and the transmembrane domains are in the outward configuration [54]. The heterodimeric ABC protein TM287/288 was crystallized in both the apo state and with nucleotide bound at NBS1: there was little difference in these two structures, the NBDs being close together and the TMs in the inward-facing conformation [55,56]. Finally, PgP was crystallized in apo state, but in this case the NBDs were wide apart and the TMs in the inward-facing conformation [57].

This would suggest that SUR cycles between an active state, in which MgATP is bound to NBS1, MgADP is bound to NBS2 and the TMs are in the outward-facing direction, and an inactive state in which the TMs are in the inward-facing direction. In all cases, the NBDs remain close together, with changes in structure between the apo and MgATP-bound states being relatively small, and a larger conformational change taking place when nucleotide is present at both NBSs. By contrast, the NBDs lie far apart in the rundown state. However, this state will not usually be accessed in the cell, where nucleotides are always present.

Although only a single SUR is depicted in figure 4, the K_ATP_ channel comprises four SUR subunits. Nucleotide binding to a single SUR does not cause channel activation—binding to at least three and probably all four subunits is required [62,63]. This suggests that Mg-nucleotide activation may fail if even a single SUR adopts the rundown configuration.

## 8. Concluding remarks

While rundown of K_ATP_ activity may at times be considered a nuisance, its study has also highlighted important mechanisms by which K_ATP_ is regulated under physiological conditions. For example, rundown due to PIP_2_ degradation clearly demonstrates the important role of anionic phospholipids in the maintenance of channel activity in the intact cell. It may even contribute to the resting ATP sensitivity of the channel. Changes in membrane phospholipids as a consequence of receptor-mediated modulation may also regulate K_ATP_ channels *in vivo.* Similarly, while the decline in Mg-nucleotide activation over time (DAMN) can be problematic for the experimenter, it suggests a new view for how the NBDs of SUR move during the gating cycle; an idea that is supported by the different structures of related ABC proteins. Therefore, while it is necessary to identify and compensate for the effects of channel rundown of DAMN in experiments on K_ATP_ channels, the underlying processes themselves have provided valuable mechanistic and physiological insights into the regulation of this most fascinating and complex ion channel.
