# Correction to ‘Running out of time: the decline of channel activity and nucleotide activation in adenosine triphosphate-sensitive K-channels’

**DOI:** 10.1098/rstb.2016.0330

**Published:** 2016-09-05

**Authors:** Peter Proks, Michael C. Puljung, Natascia Vedovato, Gregor Sachse, Rachel Mulvaney, Frances M. Ashcroft

*Phil. Trans. R. Soc. B* **371**, 20150426 (2016; Published 4 July 2016) (doi:10.1098/rstb.2015.0426)

The *x*-axis labels in figure 2*b* are incorrect. The corrected figure 2 is given below.

**Figure 2.**
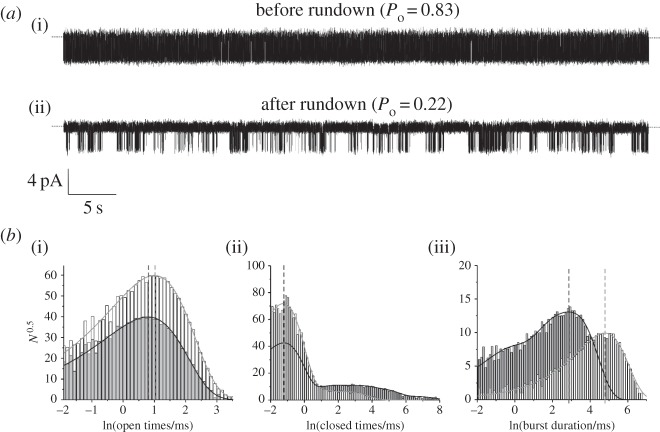
Effect of rundown on single-channel K_ATP_ channel properties. (*a*) Representative 1 min recording of single Kir6.2-G334D/SUR1 channels at −60 mV in the cell-attached configuration (*a*, *P*_o_ = 0.83) and after 5-min after patch excision (*b*, *P*_o_ = 0.22). (*b*) Distributions of channel open times (i), closed times (ii) and burst duration (iii) before (pale grey bars) and after (dark grey bars) rundown. The distributions were fitted with probability density functions that gave the following values for the individual components. Before rundown: mean open time, 2.7 ms; three apparent closed states with mean values of 0.3 ms (98.5%), 2 ms (0.8%) and 12 ms (0.7%); and a single burst state with a mean duration of 120 ms. After rundown: mean open time 2.2 ms; five apparent closed states with mean values 0.3 ms (88.9%), 3 ms (3.3%), 19 ms (4.3%), 82 ms (3.1%) and 650 ms (0.4%); and three apparent burst states with mean durations of 23 ms (66%), 5 ms (20.5%) and 0.5 ms (13.5%). Vertical lines indicate the mean values for open times, short closed times and the major burst duration component, and illustrate that rundown has no effect on the intraburst closed times, but reduces the mean open time and burst duration. The methods and solutions used are as described in [6].

